# Pasta Supplemented with *Opuntia ficus-indica* Extract Improves Metabolic Parameters and Reduces Atherogenic Small Dense Low-Density Lipoproteins in Patients with Risk Factors for the Metabolic Syndrome: A Four-Week Intervention Study

**DOI:** 10.3390/metabo10110428

**Published:** 2020-10-26

**Authors:** Rosaria Vincenza Giglio, Giuseppe Carruba, Arrigo F.G. Cicero, Maciej Banach, Angelo Maria Patti, Dragana Nikolic, Letizia Cocciadiferro, Maurizio Zarcone, Giuseppe Montalto, Anca Pantea Stoian, Yajnavalka Banerjee, Ali A. Rizvi, Peter P. Toth, Manfredi Rizzo

**Affiliations:** 1 Department of Health Promotion Sciences Maternal and Infantile Care, Internal Medicine and Medical Specialties (PROMISE), University of Palermo, 90127 Palermo, Italy; rosaria.vincenza.giglio@alice.it (R.V.G.); pattiangelomaria@gmail.com (A.M.P.); giuseppe.montalto@unipa.it (G.M.); manfredi.rizzo@unipa.it (M.R.); 2 Division of Research and Internationalization, ARNAS-Civico Di Cristina e Benfratelli, 90127 Palermo, Italy; giuseppe.carruba@arnascivico.it (G.C.); leticoccia@libero.it (L.C.); zarcone7@gmail.com (M.Z.); 3 Medical and Surgical Sciences Department, Alma Mater Studiorum University of Bologna, 40138 Bologna, Italy; afgcicero@gmail.com; 4 Department of Hypertension, WAM University Hospital in Lodz, Medical University of Lodz, 90-419 Lodz, Poland; Maciej.banach@iczmp.edu.pl; 5 Polish Mother’s Memorial Hospital Research Institute (PMMHRI) in Lodz, 93-338 Lodz, Poland; 6 Department of Diabetes, Nutrition and Metabolic Diseases, Carol Davila University of Medicine and Pharmacy, 050474 Bucharest, Romania; ancastoian@yahoo.com; 7 Department of Biochemistry, Mohammed Bin Rashid University of Medicine and Health Sciences, 505055 Dubai, UAE; Yajnavalka.Banerjee@mbru.ac.ae; 8 Division of Endocrinology, Diabetes and Metabolism, University of South Carolina School of Medicine, Columbia, SC 29203, USA; ali.abbas.rizvi@emory.edu; 9 Division of Endocrinology, Metabolism, and Lipids, Emory University School of Medicine, Atlanta, GA 30322, USA; 10 CGH Medical Center, Sterling, IL 61081, USA; peter.toth@cghmc.com; 11 School of Medicine, University of Illinois, Peoria, IL 60612, USA; 12 School of Medicine, Johns Hopkins University, Baltimore, MD 21205, USA

**Keywords:** cardiovascular risk, dyslipidemia, low-density lipoprotein cholesterol, nutraceuticals, *Opuntia ficus-indica*

## Abstract

Food supplementation with *Opuntia ficus-indica* (OFI) has been associated with a significant reduction in total cholesterol, body fat, hyperglycemia and blood pressure. Since OFI may also have antioxidant and anti-atherogenic properties, we hypothesized that its supplementation might reduce atherogenic lipoproteins, including small, dense low-density lipoproteins (sdLDL). Forty-nine patients (13 men and 36 women, mean age: 56 ± 5 years) with one or two criteria for the metabolic syndrome weekly consumed 500 g of pasta supplemented with 3% OFI extract (30% of insoluble polysaccharides with high antioxidant power) for 1 month. The full LDL subclass profile was assessed by gel electrophoresis (Lipoprint, Quantimetrix, Redondo Beach, CA, USA). After 1 month of pasta supplementation, waist circumference (*p* = 0.0297), plasma glucose (*p* < 0.0001), triglycerides (*p* = 0.0137), plasma creatinine (*p* = 0.0244), urea and aspartate transaminase (*p* < 0.0001 for each) significantly decreased. A percentage increase in larger, less atherogenic LDL-1 (*p* = 0.0002), with a concomitant reduction in smaller, denser LDL-2 (*p* < 0.0001) and LDL-3 (*p* = 0.0004), were found. LDL-4 and-5 decreased, although not significantly. This is the first intervention study suggesting that pasta enriched with an OFI extract may have beneficial effects on some metabolic parameters and the LDL particle sizes, reducing atherogenic sdLDL. Future studies will help to establish if these findings impact cardiovascular outcomes.

## 1. Introduction

Cardiovascular diseases (CVD) are the most common cause of death globally [1]. Epidemiological data indicate that CVD prevalence is steadily increasing worldwide, implying that primary prevention of this and other non-communicable diseases (NCDs), including cancer, diabetes and chronic respiratory diseases, has become a compelling goal of public health strategies. In recent years, there has been a growing interest in natural bioactive compounds contained in food that might improve cardiovascular health [2,3,4,5,6], thus presenting novel strategies for population-level reduction of CVD risk [7]. In the PREDIMED observational study, subjects in the highest quintile of polyphenol consumption had a 46% lower CVD risk compared to those in the lowest quintile [8]. This and other similar studies have provided the impetus for the exploitation of food, or parts of a food, that provide medical or health benefits, including the prevention and treatment of CVD [7,9].

*Opuntia ficus-indica* (OFI) (L.) Mill is a plant extensively cultivated in numerous regions of the world for its fruit, with a pleasant flavor and a high content of minerals, vitamins, dietary fiber, and phytochemicals. In Sicily, the plant has found a particularly favorable environment, becoming a regular feature of the natural landscape. Not only do plants grown in Sicily produce fruits that invade the markets of Northern Europe, but wild plants bring forth edible fruits very much appreciated by locals. In Central and South America, OFI is coveted for its young cladodes, known as nopalitos, which are consumed as a vegetable [10]. In recent times, various vegetative parts of OFI have been exploited for nutraceutical and health-promoting purposes (fruit, leaf and combined or unidentified *Opuntia* spp. products [11]), since they exert a plethora of beneficial effects on metabolism, oxidative stress, glucose homeostasis, total cholesterol (TC), high-density lipoprotein cholesterol (HDL-C) and low-density lipoprotein cholesterol (LDL-C) [11,12]. A meta-analysis by Onakpoya [13] shows that OFI consumption can induce significant reductions in percentage body fat, blood pressure and TC.

OFI has been recommended for a variety of diseases, including metabolic syndrome (MetS) and diabetes [14,15], and also for promoting wound healing [16]. It is clear that lipid abnormalities, including high levels of LDL-C, elevated triglycerides (TG) and low levels of HDL-C, may significantly increase the risk of coronary heart disease (CHD). LDL particle size has emerged as an important predictor of CV events and progression of CHD, suggesting that both quantity and quality of particles (particularly small, dense (sd) LDL) are essential in determining CV risk [17,18,19]. The predominance of sdLDL particles (LDL-3 to -7) has been recognized by the National Cholesterol Education Program Adult Treatment Panel III as an emerging CV risk factor [18] since, as compared to larger LDL particles, smaller LDL particles confer higher atherogenic risk due to their increased residence time in the circulation, easy penetration into sub-endothelial space, and greater susceptibility to oxidative modifications, being associated with a number of cardiometabolic conditions [20,21,22,23].

Even so, there are still no data on the effects of OFI on lipoprotein subspecies. A hypothesis of the present study was that a pasta product supplemented with an OFI extract, containing 30% insoluble polysaccharides polyphenols such as phenolic acids, flavonols and flavones, can improve the full lipoprotein profile and different metabolic parameters in patients with at least one component of the MetS in a four-week intervention study. The primary objective was to assess whether this pasta product can reduce atherogenic small, dense low-density lipoproteins, while the secondary objective was to assess whether it also improves different metabolic parameters such as body weight, waist circumference, plasma lipids and plasma glucose.

## 2. Results

Of the 49 study subjects, 63% were current smokers, 31% had hypertension, 12% were obese and dyslipidemic, and 4% were diabetic (Table 1).

After four weeks of the dietary intervention with OFI-supplemented pasta, a small but significant decrease in waist circumference was observed, while neither body weight nor body mass index (BMI) significantly changed (Table 2).

In addition to the statistically significant reduction in waist circumference (*p* = 0.0297), the dietary intervention also resulted in a modification of some biochemical plasma parameters, including plasma glucose (*p* < 0.0001), triglycerides (*p* = 0.0137), plasma creatinine, (*p* = 0.0244) and urea and aspartate transaminase (*p* < 0.0001 for each) (Table 3).

Interestingly, dietary intervention with the OFI-enriched pasta produced a significant percentage increase in LDL-1 (from 49.6 ± 0.3 to 65.1 ± 0.2, *p* = 0.0002) with a concomitant reduction in LDL-2 (from 40.1 ± 0.3 to 29.6 ± 0.2, *p* << 0.0001) and LDL-3 (from 8.3 ± 0.2 to 4.6 ± 0.1, *p* = 0.0004). Even LDL-4 and LDL-5 subclasses decreased, although the differences were not statistically significant due to the low levels of these subspecies (Table 4).

Finally, Spearman correlation analysis revealed that there were no associations between changes in sdLDL and changes in any other parameter evaluated (data not shown). Multiple regression analysis revealed that changes in all the evaluated parameters were not influenced by gender or concomitant medications (data not shown).

## 3. Discussion

Currently, few studies have analyzed the potential effects, mostly in vitro but sometimes in vivo, of OFI on lipid metabolism, glucose homeostasis and oxidative damage. In a systematic review, Onakpoya et al. [13] reported that there is no evidence from randomized clinical trials, suggesting that OFI has statistically significant effects on body weight; however, there is some indication, although not conclusive, that OFI consumption may result in a significant decrease in body fat, blood pressure, and total cholesterol. In the present study, our findings suggest that the effects of OFI are principally exerted on lipid metabolism, resulting in a significant reduction in atherogenic sdLDL. In fact, after only four weeks of intervention with OFI-supplemented pasta, large LDL-1 significantly increased by 31% and sdLDL-3 decreased by 45%. This is a beneficial effect, which was achieved after a limited period of observation and with moderate consumption of our pasta.

Mechanisms potentially underlying OFI activity, both in vitro and in vivo, remain mostly undefined. However, the different parts of OFI have been shown to contain mixtures of phenols and flavonoids, betaxanthins and betacyanins, which may be, at least in part, responsible for the observed beneficial effects through hypoglycemic, hypolipidemic actions, and antioxidant properties [24]. This has been demonstrated in studies conducted on murine aortic cells, where OFI appeared to have a dose-dependent inhibitory effect on LDL oxidation, with the maximal activity being seen at a concentration of 100 μg/mL and inducing a 50% inhibition after 18 h incubation [25]. Such a beneficial effect may be associated with the presence of quercetin, a potent flavonoid with antioxidant activity. Keller and colleagues [26] also reported that an *Opuntia* cladode powder extract significantly reduced LDL oxidation in murine endothelial cells and blocked the promotion of colon cancer development in an in vitro model of colonocytes; the authors concluded that the therapeutic potential of *Opuntia* cladodes is associated with the inhibition of oxidative stress. Moreover, Padilla-Camberos et al. [27] have indicated that an aqueous extract of OFI prevents hypercholesterolemia experimentally induced in rats through the inhibition of pancreatic lipase and that this effect can be ascribed to the polyphenolic fraction of the OFI extract.

The anti-oxidant properties of OFI are well documented [28]. Circulating sdLDL particles are prone to oxidation once trapped in the subendothelial [2,29]; by reducing sdLDL, there is less substrate available to be oxidized that, at least in part, might explain the mechanism of OFI’s anti-oxidant action. As mentioned above, the effect of cladodes was studied on LDL oxidation induced by murine endothelial cells (an in vitro model mimicking the mechanism of LDL oxidation occurring in vivo in the vascular wall). Cladode solubilized in the culture medium inhibited LDL oxidation in a dose-dependent way and the subsequent formation of macrophage foam cells, which indicates that *Opuntia* spp. could inhibit an early step in the pathway of atherogenesis [26]. This observation supports our finding that OFI reduces sdLDL. This effect of *Opuntia* spp. was attributed to inhibition of nicotinamide adenine dinucleotide phosphate (NADPH)-oxidase (NOX2), leading to the decreased generation of intracellular and extracellular superoxide anion (O2^•−^), a principal reactive oxygen species (ROS) involved in LDL oxidation [26,30]. In addition, *Opuntia* spp. inhibit the nuclear translocation of the redox-sensitive transcription factor NF-κB and reduce expression of the intercellular adhesion molecule-1 (ICAM-1) and vascular cell adhesion molecule-1 (VCAM-1) [30,31].

OFI cladodes and fruits are natural sources rich in phytochemicals, antioxidants, dietary fibers and minerals [32,33,34,35]. The major phenolic compounds found in OFI flowers and cladodes are phenolic acids (hydroxycinnamic, caffeic, ferulic and coumaric acid), flavonols (quercetin, kaempferol) and flavones (isorhamnetin and isorhamnetin derivatives) [36]. The anti-atherogenic properties of *Opuntia* stem from its high content of antioxidants (polyphenols), which also could reduce lipid oxidation and peroxidation [37], as well as from dietary fibers and proteins having lipid-lowering properties [38]. The protective effect of prickly pear *Opuntia* may also derive from pectin, a soluble fiber [39] that may reduce the intestinal absorption of cholesterol [40,41]. It has been shown that sirtuin-1 may be activated by flavonoids, which in turn activates adenosine monophosphate-activated protein kinase (AMPK)-α [42], a known master switch regulator for cell metabolism. AMPK activation increases fatty acid oxidation and carnitine palmitoyl transferase 1 (CPT1) expression [43] and reduces VLDL synthesis [44]. Flavonoids may activate protein kinase C (PKC), which promotes LDL receptor expression [45] and increased LDL-C clearance from plasma. PKC activation stabilizes LDL receptor mRNA via the c-Jun N-terminal kinase (JNK pathway) in HepG2 cells [46]. Finally, flavonoids may trigger molecular mechanisms that activate peroxisome proliferator-activated receptors (PPAR)-γ [47]. An increase in lipid metabolism and mitochondrial ATP biosynthesis has been suggested as a part of the mechanism by which polyphenols induce the expression of mRNA for enzymes that are included in lipogenesis and fatty acid oxidation (FAS and CPT1A), PPAR-γ, maintenance of the mitochondrial membrane potential (MMP) and galactose-supported ATP production [48]. Higher levels of adiponectin and a greater expression of genes regulating lipid peroxidation, carnitine palmitoyltransferase-1, and microsomal triglyceride transfer protein were measured in livers from cactus-treated animals. Furthermore, a lower postprandial serum insulin level and a greater phosphorylated protein kinase B (pAkt):Akt ratio were observed in such rats fed with cactus [49].

Cactus cladodes contain high amounts of fiber, including pectin, mucilage, lignin, cellulose and hemicellulose, and generally, these substances are able to positively influence the metabolism of lipids and glucose [50]. On the other hand, antioxidant polyphenol compounds may contribute to reduced oxidative stress, preventing free radicals from damaging biomolecules such as lipids, proteins, and DNA [51]. The high antioxidant content could also attenuate lipid peroxidation, an important risk factor in atherosclerosis [38,52]. However, the lipid-lowering properties of *Opuntia* spp. are still not well elucidated. Antioxidants block lipid peroxidation, but have usually no effect on lipid levels (except resveratrol) [3,53]. Consequently, the lipid-lowering properties of *Opuntia* spp. may rather be attributed to their enrichment with dietary fibers, as supported by data from Wolfram et al. [39] who reported that prickly pears from *O. robusta* lower cholesterol levels in hyperlipidemic nondiabetic subjects, concluding that the protective effect of *Opuntia* prickly pear may depend on the soluble fiber pectin [39]. Pectin could provoke an alteration of hepatic cholesterol metabolism without affecting cholesterol absorption [41]. Likewise, glycoprotein isolated from OFI *var. saboten MAKINO* (a variety used in Korea) exerts potent antioxidant and hypolipidemic effects [54]. It should be mentioned that the ingestion of *Opuntia* prickly pears also improves platelet function and hemostatic balance, possibly contributing to decreased atherosclerotic risk [39]. The flavonoid kaempferol or isorhamnetin from *Opuntia* extracts could suppress lipid accumulation or inhibit adipogenesis through downregulation of genes regulating adipogenesis [55,56]. Overall, both the lipid-lowering and antioxidant properties of the OFI may support its efficacy to prevent or slow the rate of atherogenesis. Dietary fibers such as pectin and mucilage [57] may be responsible for the hypoglycemic effect of *Opuntia spp*. Dietary fibers slow the absorption of glucose by increasing the viscosity of food in the gut [58,59]. Polysaccharides isolated from OFI or *O. streptacantha* also exert a hypoglycemic effect as seen in an animal model [60]. A hypothesis has been put forth that *Opuntia* spp. stimulate insulin secretion by directly acting on pancreatic beta cells [61]. *Opuntia spp*. may inhibit the development of an oxidative environment caused by hyperglycemia. As shown by Berraaouan et al. [62], the cactus pear seed oil from OFI *L. Mill*. prevents the development of alloxan-induced diabetes in animals by quenching ROS.

Consumption of fruit juice and fruit *Opuntia* naturally prevents oxidative stress and improves redox status in healthy subjects [63]. As described in detail above, *Opuntia* spp. may inhibit the early stages of atherogenesis [30], which may be further supported by reduced levels of sdLDL particles found in the present study. In this context, Budinski et al. [64] have shown that in patients with heterozygous familial hypercholesterolemia, regular consumption of *Opuntia robusta* prickly pears significantly reduces plasma levels of LDL-C, while no effect on HDL-C or TG could be observed. In our study, TC, LDL-C and HDL-C remained unchanged, while TG significantly decreased after four weeks of dietary intervention with a pasta product functionalized with OFI cladode extract. These findings may be a manifestation of the dietary intervention with OFI, considering that TG and cholesterol are different types of lipids [65]. In addition, the baseline level of TG was within the normal range, while both LDL-C and TC were high or borderline high, so it might be that longer periods of supplementation are needed to achieve the changes in cholesterol levels. Furthermore, the changes in quality of LDL-C (not in the quantity) seen in the present study should be highlighted, considering the known fact that the same level of cholesterol in two different persons can be associated with different CV risk, which depends on the prevalence of sdLDL particles [66,67,68]. This finding might be of important clinical significance, especially because it has likely been modulated by a dietary approach which remains the first line of treatment of metabolic disorders according to European guidelines [69]. However, it is clear that a firm conclusion cannot be drawn, and future studies are needed to confirm such data obtained from this pilot study. On the other hand, it has been reported that the intake of different OFI cladodes does not reduce the plasma cholesterol level [70]. This discrepancy may result from the *Opuntia* components (cladodes versus seeds) or from the diet (basal or cholesterol-enriched) [52]. In addition, it has been reported that the consumption of OFI in women resulted in a rapid increase of circulating HDL-C levels and a concomitant decrease of LDL-C and, slightly, TG [71]. Based on the previously reported increase of plasma HDL-C concentrations by OFI, it cannot be excluded that a beneficial modulation of HDL subclasses beyond what we report here in the present article on sdLDL; similarly, it cannot be excluded that there is a role for augmenting HDL particle functionality, but this remains to be further tested in future studies [72].

## 4. Materials and Methods

The study was performed at the Division of Research and Internationalization of ARNAS-Civico in Palermo, Italy (the ethical protocol code: CIVICO/19; the date: 15 February 2015), and all subjects consented to participating in the study. In total, 49 subjects aged 40–65 years (13 male and 36 female, age: 56 ± 5 years) were recruited based on inclusion and exclusion criteria. Exclusion criteria included the presence of the MetS; severe hepatic or kidney failure; major cardiovascular event(s); serious infections, such as with the Human Immunodeficiency Virus (HIV), the Hepatitis B virus (HBV) or the Hepatitis C virus (HCV); and history of cancer. Inclusion criteria consisted of the presence of one or two criteria for the metabolic syndrome, according to the American Heart Association and the National Heart, Lung and Blood Institute (AHA/NHLBI) guidelines [73]: (a) fasting glucose ≥ 100 mg/dL (or receiving drug therapy for hyperglycemia); (b) blood pressure ≥ 130/85 mm Hg (or receiving drug therapy for hypertension); (c) triglycerides ≥ 150 mg/dL (or receiving drug therapy for hypertriglyceridemia); (d) HDL-C < 40 mg/dL in men or < 50 mg/dL in women (or receiving drug therapy for reduced HDL-C); (e) waist circumference ≥ 102 cm in men or ≥ 88 cm in women. None of the participants in this study had MetS, which was defined by the concomitant presence of at least three criteria, as above. All other drugs remained unchanged throughout the study in order to minimize potential confounding effects. Before enrollment in the present study, all subjects were on stable doses of concomitant drugs for at least 4 weeks.

### 4.1. Dietary Supplementation of OFI

The dietary intervention consisted of weekly consumption of at least 500 g of a dried pasta functionalized with a soluble extract of OFI’s cladodes (replacing 500 g of the pasta commonly consumed) for a total of 4 weeks and was maintained as an add-on to the cardio-metabolic therapies already in use and at stable doses throughout the study. All participants were accustomed to Mediterranean dietary habits, according to information collected at the time of enrollment through a nutrition assessment form. They were strongly advised to keep their dietary habits and to not change their lifestyle during the study. Weekly telephone calls were made in order to ensure that. A little physical activity and sedentary lifestyle were common among the subjects. At baseline and the end of the dietary intervention, all subjects went through the following: (1) anthropometric measures; (2) psychometric tests; (3) medical examination; (4) biochemical assessment of circulating parameters, including metabolic parameters and the full spectrum of LDL subfractions. Before entering the study, participants were requested not to vary their food and/or physical activity for the entire duration of the study. All subjects had a constant consumption of durum wheat pasta, and the pasta supplied did not vary in quantity (isocaloric substitution). After participation in the study was complete, written informed consent was obtained from all participants, and the procedures adopted were in agreement with the Helsinki Declaration of 1975 as revised in 1983 and approved by the Ethics Council of the University of Palermo.

### 4.2. Pasta Preparation

OFI-supplemented pasta was obtained by the addition of 3% OFI extract (30% of insoluble polysaccharides with high antioxidant power) to flour of high quality, high-protein-content local durum wheat cultivar Pietrafitta. The 3% supplementation with the OFI extract was selected among other proportion tested (6% and 12%) as the best compromise for a pasta product having good organoleptic and sensory characteristics in terms of cooking consistency and palatability.

### 4.3. Biochemical Analyses

At baseline and after one month of supplementation with pasta enriched with OFI extract, a large number of plasma parameters were measured by routine laboratory methods, including metabolic parameters. LDL-C was calculated using the Friedewald formula.

### 4.4. LDL Subclass Analysis

Blood samples were obtained at rest after 14 h fasting overnight; blood was drawn from the subject’s antecubital vein into sterile tubes. Serum was separated and stored for subsequent analysis. Nondenaturing, linear polyacrylamide gel electrophoresis was used to separate and measure LDL-C subclasses, with the LipoPrint System (Quantimetrix Corporation, Redondo Beach, CA, USA) [74]. This method has been validated against gradient gel electrophoresis and nuclear magnetic resonance, and it is the only Food and Drug Administration-approved diagnostic tool for lipoprotein subfraction testing in the USA [18]. The electrophoresed gels were scanned to determine the relative area of each lipoprotein subfraction, and the diagnostic procedure was performed for 60 min, with 3 mA for each gel tube [75,76]. Each electrophoresis chamber involved two quality controls, and for quantification, scanning was performed with a digital scanner and a Macintosh personal computer (Apple Computer Inc, Cupertino, CA, USA) [75,76]. After scanning, electrophoretic mobility and the area under the curve were calculated qualitatively and quantitatively; LDL subclasses were distributed as seven bands (LDL-1 to LDL-7, respectively): LDL-1 and -2 as large LDL, and LDL-3 to -7 as small LDL [75,76,77].

### 4.5. Statistical Analysis

Statistical analysis was performed with the SPSS software (V.17.0 for Windows, SPSS Inc., Chicago, IL, USA). All variables were tested for normality using the Kolmogorov-Smirnov test. Data are expressed as mean (standard deviation) and as median (range), whereas categorical variables are expressed as percentages. Differences in clinical and biochemical parameters, analyzed at baseline and after one month of enriched pasta delivery in OFI, were evaluated by the paired t-test for normally distributed parameters and Wilcoxon rank test for nonparametric variables. Spearman correlation analysis was also performed to test potential associations between changes in sdLDL and changes in any other parameter evaluated. Multiple regression analysis was performed in order to reveal potential confounding effects of gender and concomitant medication (which can potentially influence outcome variables) on changes in all the investigated parameters.

## 5. Conclusions

In recent years, there has been increased clinical need for, and strong research efforts focused on, natural approaches to MetS management [78,79] and even food supplementation with OFI [80,81,82,83]. Although our study was relatively small and limited in drawing any definitive inferences, especially in view of inter-/intra-individual variability, the preliminary data presented herein is highly suggestive of a potential beneficial protective activity of OFI extract on CVD based on its effects on both lipid and glucose metabolism. To our knowledge, this is the first study investigating the impact of a pasta product supplemented with OFI cladode extract in subjects with one or two criteria for the MetS. Interestingly enough, our findings indicate that such dietary intervention seems to have positive effects on lipoproteins distribution, notably with a significant reduction of atherogenic sdLDL (−45%). Our study is relatively weakened by some potential limitations, including a limited number of study subjects, short duration of dietary intervention, variability in dietary habits of subjects, and the absence of a control group. However, statistical analyses conducted using both parametric and non-parametric tests virtually exclude the possibility that these results presented herein could have occurred randomly. In any case, furthermore extensive and longer prospective studies are required to confirm the data obtained thus far and to get significant insights into the role and underlying mechanisms of OFI in the regulation of biochemical parameters associated with CV risk. Together with the present results, it might contribute to the development of nutritional strategies, including *Opuntia* compounds.

## Figures and Tables

**Table 1:** Baseline characteristics of all patients (*n* = 49).

Age (years, mean ± SD)	55.1 ± 5.4
Women, *n* (%)	36 (73.5)
Smoking Habit, *n* (%)	30 (61.2)
Hypertension, *n* (%)	15 (30.6)
Dyslipidemia *n* (%)	6 (12.2)
Obesity (BMI > 30 kg/m^2^), *n* (%)	5 (10.2)
Diabetes, *n* (%)	2 (4.1)

**Table 2:** Changes of biometric parameters after four weeks of dietary intervention with OFI-supplemented pasta in all patients. In bold are the *p*-values that reached statistical significance.

Variable	At Baseline	After 1 Month	*p*-Value
Weight (kg)			0.8665
Mean (SD)	69.5 (14.6)	69.8 (14.1)	
Median (Min–Max)	67.8 (47.9–125.4)	69.1 (47.8–123.4)	
Waist circumference (cm)			**0.0297**
Mean (SD)	92.3 (12.3)	91.4 (10.5)	
Median (Min–Max)	92 (73–132)	91 (72–129)	
BMI (kg/m^2^)			0.8788
Mean (SD)	25.9 (3.9)	26.1 (3.7)	
Median (Min–Max)	25.3 (20.8–41.4)	25.5 (20.8–40.8)	

**Table 3:** Changes of plasma parameters after four weeks of dietary intervention with OFI-supplemented pasta in all patients. In bold, the *p*-values that reached statistical significance.

Variable	At Baseline	After 1 Month	*p*-Value
Urea (mg/dL)			**<0.0001**
Mean (SD)	32.7 (7.9)	43.3 (9.3)	
Median (Min–Max)	33 (17–51)	43 (23–68)	
Creatinine (mg/dL)			**0.0244**
Mean (SD)	0.74 (0.1)	0.72 (0.1)	
Median (Min–Max)	0.71 (0.52–1.33)	0.71 (0.43–1.25)	
Glycemia (mg/dL)			**<0.0001**
Mean (SD)	84.6 (12)	74.4 (14.2)	
Median (Min–Max)	84 (58–128)	72 (60–158)	
HbA1c (%)			0.9516
Mean (SD)	5.4 (0.4)	5.4 (0.4)	
Median (Min–Max)	5.3 (4.7–7.6)	5.3 (4.7–7.5)	
Total Cholesterol (mg/dL)			0.9620
Mean (SD)	208.3 (36.1)	209.9 (35.6)	
Median (Min–Max)	210 (115–267)	208 (129–266)	
HDL (mg/dL)			0.6672
Mean (SD)	60.7 (13.9)	60.6 (13.3)	
Median (Min–Max)	61 (30–88)	61 (30–88)	
LDL (mg/dL)			0.5274
Mean (SD)	139.3 (32.9)	139.3 (33.6)	
Median (Min–Max)	138 (65–195)	139 (78–200)	
Triglycerides (mg/dL)			**0.0137**
Mean (SD)	104.6 (53.6)	92.8 (54.7)	
Median (Min–Max)	82 (36–271)	71 (44–300)	
Aspartate transaminase (mU/mL)			**<0.0001**
Mean (SD)	28.3 (8.2)	23.7 (8.4)	
Median (Min–Max)	26 (17–63)	21 (15–62)	
Alanine transaminase (mU/mL)			0.8830
Mean (SD)	22.2 (11.4)	22.6 (13.0)	
Median (Min–Max)	18 (8–65)	19 (8–69)	
Gamma GT (U/L)			0.3855
Mean (SD)	31.5 (27.5)	33.6 (39.2)	
Median (Min–Max)	27 (11–167)	25 (11–268)	

**Table 4:** Effect of OFI-supplemented pasta on the percentage distribution of LDL subfractions. Data are expressed as mean ± SD. In bold, the *p*-values that reached statistical significance.

Variable	At Baseline	After 1 Month	*p*-Value
LDL-1	49.6 ± 0.3	65.1 ± 0.2	**0.0002**
LDL-2	40.1 ± 0.3	29.6 ± 0.2	**<0.0001**
LDL-3	8.3 ± 0.2	4.6 ± 0.1	**0.0004**
LDL-4	1.3 ± 0.1	0.6 ± 0.0	0.2987
LDL-5	0.7 ± 0.1	0.1 ± 0.0	0.3223
LDL-6	-	-	*-*
LDL-7	-	-	*-*
